# Psychological distress in spouses of somatically Ill: longitudinal findings from The Nord-Trøndelag Health Study (HUNT)

**DOI:** 10.1186/s12955-014-0139-7

**Published:** 2014-09-09

**Authors:** Ingrid Borren, Kristian Tambs, Kristin Gustavson, Jon Martin Sundet

**Affiliations:** Norwegian Institute of Public Health, Division of Mental Health, PO Box 4404, Nydalen, N-0403 Oslo, Norway; Department of Psychology, University of Oslo, PO Box 1094, Blindern, N-0317 Oslo, Norway

**Keywords:** Caregiving, Spouse, Somatic illness, Mental health, Longitudinal, Population-based, Norway

## Abstract

**Background:**

Studies of caregiver burden and somatic illness tend to be based on relatively small, clinical samples. Longitudinal, population based studies on this topic are still scarce and little is known about the long-term impact of partner illness on spousal mental health in the general population. In this study we investigate whether spouses of partners who either have become somatically ill or cured from illness in an 11 year period - or who have long-term illness - have different mental health scores compared to spouses of healthy partners.

**Methods:**

Approximately 9000 couples with valid self-report data on a Global Mental Health (GMH) scale and somatic illness status were identified. The diagnoses stroke, angina pectoris, myocardial infarction and severe physical disability, were transformed into a dichotomous ‘any illness’-scale, and also investigated separately. Analyses of variance (ANOVA) stratified by sex were conducted with spousal GMH score at follow-up (1995–97, T2) as the outcome variable, adjusting for spousal GMH score at baseline (1984–86, T1) and several covariates.

**Results:**

Results showed that male and female spouses whose partners had become somatically ill since T1 had significantly poorer mental health than partners in the reference category, comprising couples healthy at both time points. Further, female spouses of partners who had recovered from illness since T1 had significantly better mental health than controls. Of the somatic conditions, physical disability had the most significant contribution on spousal GMH, for both sexes, in addition to stroke on male spouses’ GMH. The effect sizes were small. Some of the loss of spousal mental health seems to be mediated by the ill persons’ psychological distress.

**Conclusion:**

The occurrence of partner illness during the follow-up period affect the mental health of spouses negatively, while partner recovery appeared to be associated with improved mental health scores for female spouses. Of the measured conditions, physical disability had the largest impact on spousal distress, but for some conditions the distress of the ill person mediated much of the loss of mental health among spouses.

## Introduction

Primary caregivers of physically disabled or persons suffering from some kind of life-threatening or chronic disease are essential supporters of the patient. The burden of taking care of ill or disabled individuals falls increasingly upon family members in many countries, including Norway. For instance, the trend towards early discharge from hospital for patients leaves the spouse or relatives with more caregiving tasks in the home [1–3].

It has long been established that taking care of a somatically ill family member can impose psychological distress and reduced subjective well-being for the caregiving family members [4–7] and more so with increasing patient disability [8]. In addition to causing lowered mental health in caregivers, it has also been found that caring for ill family members is associated with reduced physical health in caregivers [9–11]. Caregivers of physically disabled living at home are faced with continuous strains both physically and mentally, as this condition requires abundant assistance with various tasks [12,13]. In many cases the long-term aspect of this situation may add to the perceived burden.

Individuals who have suffered a myocardial infarction (MI), or who experience recurrent angina attacks, normally live at home after these incidents and their family members have been found to experience emotional distress, caregiver dysfunction and post-MI stress [14,15]. Findings from a recent study indicate that not only caregiving tasks impose caregiver strain, but the very exposure to spousal suffering may contribute to psychiatric and physical morbidity [16]. Likewise, it has been found that caregivers of stroke patients tend to report poorer mental health and prominent depressive symptoms [17–20]. The severity of the patient disability and immobility has also been found to be associated with heavier caregiver burden [8,12].

Population-based studies have shown that the amount of time spent caring progressively increases the risk of onset of distress, and that the levels of distress and depressive symptoms raise with higher time commitment to informal care [21,22]. Spousal caregivers of physically disabled providing 36 hours of care or more weekly have been found to be 6 times more likely than non-caregivers to experience depressive or anxious symptoms [23]. The transition periods in and out of the caregiver role have in addition been found to have the most adverse effect on mental health of caregivers with much involvement [22].

Gender differences have been a topic of much interest in this field and female gender has repeatedly been reported to be a risk factor for caregiver burden [24–28]. A previous review [29] also confirmed that women display more psychiatric symptomatology like anxiety and depression than do men. This review suggested that these findings were attributable to caregiving and that this may be due to the fact that women respond differently to all stages of the stress process than do men, and that they engage more in caregiving tasks and spend more time on this. However, this interpretation remains uncertain, as Pinquart and Sorensen reported in their meta-analysis [30] that the gender differences disappeared when statistically controlling for gender differences in stressors and resources.

Much of the existing knowledge on caregiver burden has been obtained from cross-sectional studies. Although they provide useful information about associations on this topic, longitudinal studies are needed to examine the mechanisms and the dynamics of these relationships over time. Longitudinal studies may provide additional information about the relationship between spousal distress and caring for a spouse with long-term illness or experiencing a transition into or out of the caregiver role. Previous studies investigating caregiver burden among relatives of somatically ill individuals have, however, mainly been selected, clinical studies which in most cases have focused on one, specific illness i.e. [4,13,14,31–33].

The current study avoids some of the mentioned methodological shortcomings of previous studies, as several different somatic illnesses have been studied in a large, population-based sample. Our aim is thus to investigate the effects of an overall score of partners’ somatic illness on spousal psychological distress – both long-term and more recent cases - over an 11 year follow-up period. Each of the somatic conditions will also be analysed separately in order to investigate whether different conditions generate different levels of distress. Based on previous research we hypothesize that the occurrence of stroke and cardiac diseases may have a detrimental effect on caregivers’ mental health. It is also likely that both the long-term and more recent cases of physical disability may have a substantial impact on spousal distress as this condition tends to be chronic and require much care. Also, the participants included in this group report severe disability, and previous research has found a dose–response relationship between the degree of disability and the degree of spousal distress [8]. All analyses will be stratified by sex and in line with the previously mentioned findings we hypothesize that female spouses may display more distress than male spouses potentially due to more involvement in caregiving tasks.

## Methods and materials

### Design

A longitudinal design was applied in the current study to investigate whether change in the ill partner’s health status would affect the mental health of the spouse. Our aim was to explore the association between the affected partners’ status as ill or healthy at baseline (T1) and follow-up (T2), and the spouses’ level of distress at T2, adjusting for spousal distress at T1. The affected partner’s case status was classified into four categories; the reference category comprised those who were healthy at both T1 and T2 (well/well) and the other three groups were cases at T1 but not T2 (ill/well), cases at T2 but not T1 (well/ill), and ill at both T1 and T2 (ill/ill).

### Measures

Information about *somatic illness* was obtained from Q1 where the participants reported if they had ever received one or more of the following diagnoses from a physician: myocardial infarction (MI), angina pectoris or stroke. We also included in the illness category respondents who perceived themselves to be severely physically disabled (‘heavy impairment of motor abilities’ on a three point scale as a result of either injury or illness). Positive response to one of these items in 1984 and negative in 1995, would hence assign the participants to the ill/well category, and vice versa. The distribution of illnesses among men and women in our sample is reported in Table 1. All diagnoses, as well as physical disability, were computed into a scale called ‘any illness’. We also investigated the effect of each somatic condition on spousal distress separately.

**Table 1:** **Distribution of educational level, occupational status and somatic conditions in the study sample**

	**Males**		**Females**	
	**N**	**%**	**N**	**%**
	Educational level			
Elementary school (7–10 yrs)	3578	39.7	4534	50.4
High school (1–2 yrs)	3436	38.1	2674	29.7
High school (completed, 3 yrs)	358	4.0	420	4.7
College/university (<4 yrs)	894	9.9	840	9.3
College/university (>4 yrs)	751	8.3	527	5.9
	Occupational status at T2, 1995-97			
Paid work	4186	46.5	4936	54.7
Self-employed	2031	22.6	952	10.6
Homemaker	33	0.4	2098	23.3
Student/military service	86	1.0	111	1.2
Unemployed	137	1.5	200	2.2
Social security benefit	3309	36.8	2580	28.6
	Somatic health status at T1 and T2, 1984-1995			
Somatic condition				
***Disability***				
Long-term (T1 and T2)	125	1.4	109	1.2
Recovered (ill T1, well T2	183	2.0	115	1.3
Become ill (well T1-ill T2)	492	5.5	441	4.9
No disability T1-T2	8195	91.1	8352	92.8
Total	8994	100	8994	100
***Stroke***				
Long-term (T1 and T2)	38	0.4	22	0.2
Recovered (ill T1, well T2	7	0.1	9	0.1
Become ill (well T1-ill T2)	214	2.4	121	1.3
No stroke T1-T2	8736	97.1	8865	98**.**6
Total	8994	100	8994	100
***Myocardial infarction***				
Long-term (T1 and T2)	150	1.7	11	0.1
Recovered (ill T1, well T2)	11	0.1	0	0.0
Become ill (well T1-ill T2)	476	5.3	104	1.2
No MI T1-T2	8358	92.9	8902	98.9
Total	8994	100	8994	100
***Angina***				
Long-term (T1 and T2)	192	2.1	67	0.7
Recovered (ill T1, well T2)	38	0.4	21	0.2
Become ill (well T1-ill T2)	622	6.9	243	2.7
No angina T1-T2	8143	90.5	8686	96.5
Total	8994	100	8994	100

#### Index of psychological distress

The Nord-Trøndelag Health Study 1 (HUNT 1, see “Sample” section) did not include an established measure of mental health, therefore we had to generate a scale from available items pertaining to mental health. The outcome variable measuring spousal distress is a combination of nine items which were included in the questionnaires in both HUNT 1 (at T1) and HUNT 2 (T2): *‘Do you often feel lonely?’* (5 response categories, ‘very often’-‘never’), ‘*During the last month, have you suffered from nervousness (felt irritable, anxious, tense or restless)?*’ (4 response categories, ‘almost all the time’-‘never’), ‘*Do you feel for the most part strong and fit or tired and worn out?’* (7 response categories, ‘very strong and fit’-‘very tired and worn out’), ‘*During the last month, have you had any problems falling asleep or sleep disorders?’* (4 response categories, ‘almost every night’-‘never’), ‘*Do you suffer from any long-term illness or injury of a physical or psychological nature that impairs your functioning in your everyday life? (Long-term means that it has lasted or will last for at least one year). If YES, would you describe your impairment as slight, moderate or severe? [Among different types of impairment] Impairment due to mental health problems*.’ (3 response categories, ‘slight’-‘severe’), ‘*Do you by and large feel calm and good?’* (4 response categories, ‘almost all the time’-‘never’), *‘Thinking about your life at the moment, would you say that you are by and large satisfied with life or that you are mostly dissatisfied with your life?’* (7 response categories, ‘very satisfied’-‘very dissatisfied’), ‘*Would you say you are usually cheerful or downhearted?’* (6 response categories, ‘very downhearted’- ‘very cheerful’) ‘*How often have you taken tranquilizers, sedatives or sleep medication during the last month*?’ (4 response categories, ‘daily’-‘never’). A summative, standardized index was generated on the basis of these 9 items. The items were empirically selected based on a stepwise multiple linear regression analysis of available data from a sub-sample from HUNT described elsewhere [34] containing both the ordinary HUNT 1 items and the well-established Hopkins Symptom Check List (SCL)-25, tapping symptoms of anxiety and depression. The SCL-25 score was used as dependent variable in the regression analyses and items from the ordinary HUNT 1 questionnaire as predictor variables. The nine items were selected because they independently contributed to the variance in SCL-25. All nine items correlated 0.50 or higher with the SCL-25 score. The correlation between our summative indicator, called Global Mental Health Index (GMH) and the SCL-25 score was 0.83. The construction of the GMH is described in more detail elsewhere [35]. Cronbach’s alpha for the GMH scale at T1 and T2 was virtually identical; about .82 and .83, respectively, for both sexes. High GMH values express distress.

#### Control variables

We adjusted for the caregiving spouses’ mental health (GMH) at T1, and also age, education (measured on a 5 point scale ranging from ‘elementary school’ (7–10 years) to ‘university’ (4 years or more), the caregiving spouses’ somatic illness status at T2 (a sum score similar to the previously described measure of somatic illness utilized as predictor), and the affected partners’ mental health (GMH) at T2.

### Sample

The current study is based on longitudinal self-report data from The Nord-Trøndelag Health Study, HUNT. Nord-Trøndelag county comprises 3% (133,045 persons in 2011) of the Norwegian population. The county is fairly representative of the population of the rest of the country in terms of geography, economy, industry, sources of income and age distribution, although slightly less urban and with somewhat lower educational attainment. The entire adult population (aged 20 or above) of the county was invited to participate in HUNT. The current study is based on data from the first two waves of data collection, namely HUNT 1 (1984–86, T1) and II (1995–97, T2). Those who were invited participated in a health examination and were requested to complete two questionnaires. The first, Q1, was returned at the screening site and the other (Q2) was completed at home and returned by pre-paid mail. 85,125 persons, 88.1% of the total adult population, participated in HUNT 1 and returned Q1. Of these, 75.1% returned Q2. Out of the 94,194 individuals who were invited to HUNT 2, a total of 92,936 were eligible for participation. 71.2% (66,140) of these participated. 47,286 individuals participated both at T1 and T2. The HUNT 2-sample is described in detail elsewhere [36].

Personal identification numbers for married and cohabiting couples made anonymous by encryption were obtained from the governmental statistics agency, Statistics Norway, and these were matched with HUNT-data in order to identify registered couples. For the purpose of the current study, only couples who were married or cohabitants at T1 and still together at T2 and of which both partners had answered both Q1 and Q2 at both T1 and T2 were included in the analyses. We thus identified 9404 mixed-sex pairs. Of these only couples having complete data on psychological distress, somatic illness and all covariates were included in the analyses, which resulted in 8994 couples. This number corresponds to 22.1% of the invited couples at T1 and 28.4% at T2. Age at T1 ranged from 21 to 83 years for female participants (mean age 44.3, SD 11.9) and 22–88 for males (mean age 47.3, S.D 12.2). At T2 age ranged from 32 to 94 years for females (mean age 55.5, SD 11.9) and from 33 to 99 years for males (mean age 58.5, SD 12.2). For educational and occupational information, see Table 1.

### Statistical analysis

Repeated measures ANOVA (SPSS General Linear Models, UNIANOVA) was conducted with GMH at T2 as the outcome variable and the partners’ belonging to one of the four case categories was entered as factor, adjusting for the spouses GMH at T1. In the second model age and education were controlled for in addition to GMH1. In the third model, the somatic health status of the caregiver was added as covariate in addition to the previously mentioned three, and in the last model the affected (ill) partners’ GMH at T2 was entered together with the other covariates. Due to the files’ double entry structure, all analyses were stratified by sex in order to avoid statistical dependency between observations. There were 8994 pairs with complete data. All three illness categories were compared to the reference group. Analyses were conducted both when the somatic conditions were collapsed into one illness variable and separately for each type of somatic condition. The dependent variable – GMH at T2 – was standardized before performing the analysis. Mediation tests were conducted by means of the traditional Baron and Kenny steps [37] and Sobel tests [38].

### Treatment of missing values

SPSS Missing Value Analysis (MVA), expectation maximization (EM) was used to replace missing values for participants having completed at least half of the GMH items. Missing values were thus reduced from 5.8% to 0.1% for GMH at T1. At T2 people aged 70 years or older completed a questionnaire version in which one of the GMH-items was omitted. In total, including missing values caused by this missing item in the 70+ questionnaire version, 32.8% missed one or more items. However, 24.7% had only one item missing, and most of those were among the respondents completing the questionnaire version with the reduced set of items. Accordingly the actual number of subjects with missing values was 17.6%. This number was reduced to 0.2% missing for GMH at T2 after imputation. Data sets with other missing values were omitted from the analyses.

### Ethics

The HUNT studies are approved by the Data Inspectorate of Norway and by the Regional Committee for Medical Research Ethics. Data were anonymized before made available to the researchers, and all data are treated according to guidelines from the Norwegian Data Inspectorate. Participation is based on informed consent.

## Results

### Male spouses

Table 2 shows that male spouses of persons who had become ill since T1 had poorer mental health than the reference group (healthy partners at both times), when spousal GMH at T1 was the only control variable. When controlling for age, education and the spouses’ own somatic illness status, these findings prevailed. However, when additionally adjusting for the female affected partners’ GMH score at T2 the effect was no longer significant. A mediation test exploring whether female distress mediates the effect of female illness on male distress was performed and yielded significant results.

**Table 2:** **Male spouses’ symptoms of distress related to illness in female partner**

**Type of illness**	**Model 1**				**Model 2**			**Model 3**			**Model 4**		
	**N**	**b**	**(CI)**	**p**	**b**	**(CI)**	**p**	**b**	**(CI)**	**p**	**b**	**(CI)**	**p**
**Any illness**													
Ill 84-ill 95	209	.10	(−.01- .22)	.056	.10	(−.01- .21)	.075	.09	(−.02-.20)	.091	.01	(−.10- .12)	.913
Ill 84- well 95	119	.12	(−.02- .26)	.099	.11	(−.03- .26)	.122	.10	(−.05- .24)	.182	.05	(−.09- .20)	.458
Well 84-ill 95	731	.10	(.04- .16)	.001***	.19	(.04- .16)	.001***	.09	(.03- .15)	.005**	.01	(−.06- .07)	.828
Overall p-value				.001***			.003**			.010*			.900
**Physical disability**													
Ill 84-ill 95	109	.12	(−.03- .27)	.103	.12	(−.03- .27)	.107	.11	(−.03-.26)	.132	.01	(−.14- .16)	.859
Ill 84- well 95	115	.10	(−.05- .25)	.174	.09	(−.05- .24)	.212	.08	(−.07- .22)	.307	.03	(−.12- .17)	.720
Well 84-ill 95	441	.09	(.01- .16)	.027*	.08	(.01- .16)	.043*	.06	(−.02- .14)	.118	-.04	(−.12- .03)	.268
Overall p-value				.030			.052			.145			.692
**Stroke**													
Ill 84-ill 95	22	.03	(−.30- .36)	.994	.02	(−.31- .35)	.997	-.01	(−.31-.33)	.973	-.06	(−.39- .26)	.696
Ill 84- well 95	9	.04	(−.47- .56)	.917	.06	(−.45- .57)	.855	.10	(−.41- .60)	.732	.05	(−.46- .54)	.873
Well 84-ill 95	121	.16	(−.02- .30)	.030*	.16	(.02- .30)	.025*	.13	(−.01- .27)	.070	.07	(−.07- .21)	.310
Overall p-value				.195			.170			.336			.717

Note: Couples with affected partners well at both points in time (N = 7935 male spouses) form the reference groups.

b show adjusted mean deviations from spouses of persons without somatic illness in standard deviation units.

New separate analyses were conducted for each separate type of illness.

Model 1: Adjusting for spouse’s mental health at T1.

Model 2: Adjusting for spouse’s mental health at T1, age, and education.

Model 3: Adjusting for spouse’s mental health at T1, age, education, and spouse’s somatic illness status at T2.

Model 4: Adjusting for spouse’s mental health at T1, age, education, spouse’s somatic illness status at T2, and the affected partner’s mental health (GMH) at T2.

* = p < 0.05; **p = <0.01; ***p < 0.001.

The analyses of the specific illness conditions showed that men who had experienced that their partners had become physically disabled from T1 to T2 reported significantly more change in distress compared to the reference group, adjusting for spousal GMH at T1, age and education. Additionally controlling for the male spouses’ own somatic health reduced the effects of the affected partner’s physical disability to a non-significant level.

Investigating the effect of stroke in affected female partners yielded no overall significant effect of the model, but when controlling for the caregivers’ distress at T1, age and education, those who had a female partner who had suffered a stroke since T1 had significantly poorer mental health than the reference group. The effect was no longer significant when adjusting for spousal somatic health. Effect sizes in terms of adjusted mean differences between each of the affected groups and the reference (well-well) group are all quite moderate, for model 3 typically around 0.1 standard deviations.

No significant effects on male spousal mental health could be detected for myocardial infarction or angina in female partners.

### Female spouses

As shown in Table 3, when only adjusting for the spouse’s own GMH at T1 female spouses of men who had changed illness status from well to ill from T1 to T2 had significantly worse mental health compared to the reference category. The same was the case for those who had partners who were ill at both T1 and T2. However, when additionally controlling for age and education (from model 2 on) these effects were no longer significant. Instead the results from this model showed that the female spouses in the ill-well group had a significant improvement of mental health from T1 to T2, compared to the reference category. This effect prevailed when adjusting for all the other covariates.

**Table 3:** **Female spouses’ symptoms of distress related to illness in male partner**

**Type of illness**	**Model 1**				**Model 2**			**Model 3**			**Model 4**		
	**N**	**b**	**(CI)**	**p**	**b**	**(CI)**	**p**	**b**	**(CI)**	**p**	**b**	**(CI)**	**p**
**Any illness**													
Ill 84-ill 95	459	.09	(.01- .17)	.028*	.02	(−.06- .11)	.557	-.01	(−.09-.07)	.888	-.08	(−.16- .01)	.057
Ill 84- well 95	142	-.10	(−.24- .04)	.154	-.16	(−.29- -.02)	.028*	-.16	(−.30- -.03)	.020*	-.21	(−.34- -.07)	.003**
Well 84-ill 95	1211	.08	(.03- .13)	.003**	.04	(−.02- .09)	.186	.04	(−.02- .09)	.181	-.03	(−.08- .03)	.325
Overall p-value				.001***			.061			.050			.007**
**Physical disability**													
Ill 84-ill 95	125	.12	(−.03- .27)	.113	.06	(−.09- .21)	.414	.03	(−.11-.18)	.675	-.10	(−.24- .05)	.185
Ill 84- well 95	183	-.12	(−.25- -.01)	.046	-.18	(−.31- -.06)**	.003	-.19	(−.31- -.07)**	.002	-.24	(−.36- -.12)***	.000***
Well 84-ill 95	492	.19	(.11- .26)***	.000	.15	(.08- .23)***	.000	.14	(.06- .21)***	.002	.02	(−.06- .10)	.631
Overall p-value				.001***			.001***			.001***			.001**

Note: Couples with affected partners well at both points in time (N = 7182 female spouses) form the reference groups.

b show adjusted mean deviations from spouses of persons without somatic illness in standard deviation units.

New separate analyses were conducted for each separate type of illness.

Model 1: Adjusting for spouse’s mental health at T1.

Model 2: Adjusting for spouse’s mental health at T1, age, and education.

Model 3: Adjusting for spouse’s mental health at T1, age, education, and spouse’s somatic illness status at T2.

Model 4: Adjusting for spouse’s mental health at T1, age, education, spouse’s somatic illness status at T2, and the affected partner’s mental health (GMH) at T2.

*=p < 0.05; **p = <0.01; ***p < 0.001

When analysing the specific diagnoses separately, the only condition with a significant effect on female spouses’ psychological distress was physical disability. Table 3 shows that women whose partners had become physically disabled from T1 to T2 reported significantly more distress than at T1, compared to the reference group. Further, those whose partners had recovered from physical disability between the two times reported significantly less distress than at T1, compared to this group. The former effect remained significant when adjusting for age, education and the spouses’ own somatic health status. When additionally controlling for the affected partners’ GMH, only the positive effect of having a recovered partner remained significant. A mediation test investigating whether male partners’ distress mediates the effect of male disability on female spouses’ distress, yielded non-significant results.

The other illnesses yielded no significant results for female spouses. Like with male effect sizes, effect sizes in female spouses were absent or moderate. The effects of having a physically disabled husband were stronger than trivial, however, with an improvement of around 0.2 standard deviations in wives of husbands who had recovered and around 0.15 standard deviations more distress in wives of husbands who became disabled between T1 and T2.

## Discussion

The current paper adds to the previous knowledge in the field of caregiving within somatic conditions by longitudinally investigating the relative impact of several different conditions in a large population-based sample. To the authors’ knowledge such a comparison of illnesses in this kind of data has not previously been made. The results bring insight about what types of conditions that most strongly are related to spousal distress, and how changes in somatic illness in one partner may be linked to changed mental health in the other partner over time. As mentioned in the introduction, studies of caregiver burden tend to be based on small-scale, clinical studies which separately can tell little about the true effect sizes, and in which publication bias may have somewhat inflated the average effect sizes. It is important and of great value to explore these associations using data from one of the largest population based health studies ever conducted, that also had generally high response rates. Also the opportunity to follow this development in couples for as much as 11 years is rare.

### Changed somatic illness status from well to ill

Our study shows that the more recent occurrence of partner’s physical morbidity may be associated with more psychological distress in the caregiving spouse, for both men and women. This is in accordance with previous studies in this field [7,16,20] and is what would be expected, considering the fact that the transition of one partner into the role as patient necessarily will affect many aspects of the daily life for the spouse. It might both cause practical challenges (see for instance [39]) and evoke negative feelings, like fear of loss, worrying about the future, anxiety and grief in addition to the emotional burden of seeing the suffering of their partner [16,20,40].

Even though the effect sizes are small, the actual importance of the findings is context-specific. As partner’s somatic illness is something that affects a substantial proportion of the population each year, the amounts of money spent by society on caregiver’s absence from work due to caring tasks may be of considerable size in total: It has been found that caregiving reduces paid work hours for middle-aged women by 41% [41]. Numbers from the U.S. indicate that 1/5^th^ (17%) of all workers are caregivers and that losses to U.S. business productivity related to informal caregiving have been estimated to as much as $ 33.6 billion per year for full-time employees providing informal care [42].

Looking at the specific somatic conditions, it is clear that the occurrence of *physical disability* in the affected partner between T1 and T2 has a significant effect on the caregiving spouses’ level of distress, for both male and female partners. This is in accordance with previous findings concluding that increases in spousal impairment in general are related to poorer mental health outcomes over time [12]. A meta-analysis by Pinquart & Sorensen [43] concluded that physical impairment has a stronger relationship to burden for spouse caregivers than for adult children. Having a partner who becomes dependent will naturally generate a bigger workload for the healthy spouse and cause more practical problems in everyday life in relation to immobility and need for assistance with daily tasks [7,8]. The fact that physical disability in many cases also is a permanent condition may be hard to accept, as it may bring about major life-style changes for the family and possibly alter the dynamics and the balance of an intimate relationship [44].

The occurrence of *stroke* in affected female partners since T1 had a significant effect on male spouses. No corresponding effect on female partners of stroke patients could be detected. Generally, caregivers of stroke patients tend to report poorer mental health and prominent depressive symptoms [17–20]. Female spouses have previously been found to have an increased risk of burden and poorer outcome on several measures one year post stroke [28,45]. However, although female spouses indeed reported more symptoms of anxiety and depression shortly after a stroke occurred, some studies have found that men fare worse than women in later follow-up studies [46,47]. This may imply that female spouses adjust more successfully to the changes linked to their partners’ illness in time than do male spouses.

None of the *heart conditions* were found to represent any significant effects on either male or female caregivers. This may seem puzzling, given that especially MI is a serious condition which is among the most common causes of death worldwide [48] and since the risk of reinfarction is elevated after a primary infarction [49]. The element of repetition in angina attacks could potentially also be a stressor as it may keep reminding the spouses of the potential threat of an eventual MI, and further, it might be stressful experiencing the partner struggle with recurring heart pains. Earlier research has concluded that caregiver burden and distress are clearly present in these diseases [50,51]. However, most research on caregiver burden and somatic illness is carried out on clinical samples where participants are selected because they are in the midst of the course of illness. In this study, participants have, on the other hand, reported that they at “some point in time” have been diagnosed with one of the relevant conditions. It is reasonable to assume that incidents of illness dating years back do not have the same impact on caregivers as more recent cases, as the affected person might have fully recovered. Also, many patients with a heart condition function well after medical procedure and without any particular symptoms, thus, a strong impact on caregivers’ distress for a long period after the incident is perhaps unlikely.

### Changed somatic illness status from ill to well

It seems to have a positive effect on the mental health of female spouses to have a partner who has recovered from illness since T1. These women displayed a beneficial change in mental health status from T1 compared to the reference group. As shown in the results section, this group basically consists of those with physically disabled partners only, as none of the other diagnoses showed significant results in female spouses. This condition is probably the most physically and practically challenging in regard to immobility and need for assistance (as included respondents have reported to have “heavy impairment of motor ability”), hence we may assume that the workload on the spouses has been particularly heavy. The decreased level of distress may well reflect appreciation of reduced workload and obligations, as well as of their partner’s improved situation in general. Improved mental health after patient recovery or nursing home admission has previously been documented [47,52]. Thus, both the negative effects of the occurred illness and the positive effect of recovery tell us that the presence of somatic conditions is a factor influencing the mental health of the spouse. These findings do not, however, support the previously mentioned findings that transitions also out of the caregiver role may have detrimental effects [22]. Anyhow, some of the possible negative transition-effects may have worn off in our study, as the time span between T1 and T2 is quite large.

### Somatic illness at both measurement times

Hardly any significant effects could be detected in this group. Female spouses who had an ill partner at both measurement times experienced significantly more distress in the initial model, whereupon the effect disappeared when adjusting for age and education. No significant effect could be detected among the spouses in this group for other illnesses. This might perhaps be expected as their situation has been unchanged. A potential adjustment to the situation could hypothetically yield a decreased level of distress, but if the spouses become gradually more exhausted due to constant pressure (“the wear and tear hypothesis”, i.e. [53]), or if the illness progresses in severity, an increment in distress should be observed. None of those effects were found, maybe because an effect of adjustment on the one hand and exhaustion and illness progression on the other counteract each other.

### The mediating effect of the affected partner’s mental health

Repeatedly throughout our analyses the effect of somatic illness has tended to disappear when controlling for the ill partner’s *level of distress*, which in general may imply that the effect of illness on spousal distress to some extent is mediated by the ill partner’s level of distress. In other words, how the ill partners cope with their situation may be even more important to their spouses than the effect of the illness per se. Mediation tests were performed, and for male spouses of female partners who have become ill since T1, it was found that the wife’s GMH totally mediated the effect of her illness. This finding supports previous studies concluding that the ill partner’s psychological distress is especially influential on the other partner in times of illness when they naturally are more prone to become depressed and distressed themselves i.e. [16,54]. This was also demonstrated by Kurtz and colleagues [4], who found patient depression to predict caregiver depression, and by Ruiz and colleagues [55] who found caregivers’ level of neuroticism pre-surgery to predict patients’ post-surgical depression, and vice versa. In cases where the somatic condition is not curable, interventions to relieve psychological distress in the affected partner may also relieve much of the caregivers’ distress, which to a large extent seems to be mediated through the ill persons’ distress.

### Methodological issues

The current study has several strengths. First of all it is based on a large and representative sample with high response rates, and the large sample sizes allow even smaller effects that could be of importance to be detected. The HUNT study is one of the largest health studies internationally, and there is a lack of population-based studies in this field. The longitudinal design of our study is a great strength as it permits us to discriminate between groups of caregivers whose partners (presumably) have long-term illness and those whose partners have become ill – or have recovered – more recently. Even though the Global Mental Health measure is not a standard scale, it correlates quite highly with the well-established SCL-25 scale and it has also been used in previous studies i.e. [35]. Further, it is positive that the participants were not aware of the purpose of this study. Not asking directly about caregiver burden may prevent biased and exaggerated responses.

However, there are certain methodological issues that need to be addressed. Due to the relatively high attrition rate when selecting our sample (both partners had to participate at both T1 and T2), the generalizability of the results may have been reduced.

Further, due to the way some of the questions in the questionnaire are phrased, discriminating between who are ill and who have recovered is in some cases difficult (‘Do you have or have you ever had (diagnosis)’. The exception is physical disability, where the items are phrased in a way that captures current cases. This should be kept in mind when interpreting the results. If these groups of cases could have been more reliably categorized as ill or well at T1 and T2 we might have revealed larger effects of partner’s illness than what we have found. For the persons belonging to the ill-ill group potentially systematic effects may have been attenuated, as some of these persons may report illnesses far back in time which may no longer have any effect (due to the possibility of misclassifications of persons from the ill-well group to the ill-ill). Strictly, for people with cardiovascular illness and stroke there should have been no cases in the ill-well group because of the previously mentioned phrasings in the questionnaire items (‘..ever had’ the illness). In the relatively few cases where the participants still have reported having had stroke, MI or angina at T1 but not at T2, this is probably associated with an improvement in their health situation. Still, the findings for this group are less reliable. The vast majority of the ill-well cases for ‘any illness’ suffered from disability, however, so the ill-well results for any illness as well as for disability are probably not severely biased by misclassification.

The results based on the comparison between the ‘well-ill’ and the reference (‘well-well’)- group are probably the most reliable, even though it may be problematic that there is about 11 years between the two waves of data collection. The illness may in some cases have occurred shortly after T1, and some effects thus may be attenuated (the exception being physical disability). If so, it is reason to believe that our findings reflect underestimated effects of spousal distress and that the effects among the spouses of the more severely ill groups may actually be larger than shown in our study. Also, it is possible that the most severely ill part of the population (both somatically and psychologically) did not participate in the study, and hence that the sample is “healthier” than the rest of the population. This might affect the generalizability of the results. Results from a recent attrition study of HUNT II, using register data and data from HUNT I, suggest that selection towards good mental health is quite moderate [56], but selection towards good somatic health may still be a threat.

## Conclusions

The current study adds to our previous knowledge by investigating the impact of several somatic conditions on spousal mental health using a large scale population-based data material. Male and female spouses having partners who had become somatically ill during the period from T1 to T2, had a significantly poorer course of mental health than partners of persons healthy at T1 and T2. Of the specific somatic conditions, the occurrence of partner’s physical disability had the most significant contribution on spousal distress, for both sexes. In addition, the occurrence of stroke in female spouses since T1 had a negative impact on male spousal distress. Much of the loss of mental health appears to be mediated by the affected partners’ own psychological distress. Conversely, female spouses of partners who had recovered from physical disability since T1 had significantly better mental health than the reference category.

The effect sizes were small, however, and our quite small estimates - although probably somewhat attenuated - should be interpreted with some optimism: most people with somatically ill partners appear to cope quite well with their caregiver burden.
